# Tissue Conditioner Incorporating a Nano-Sized Surface Pre-Reacted Glass-Ionomer (S-PRG) Filler

**DOI:** 10.3390/ma14216648

**Published:** 2021-11-04

**Authors:** Watcharapong Tonprasong, Masanao Inokoshi, Muneaki Tamura, Motohiro Uo, Takahiro Wada, Rena Takahashi, Keita Hatano, Makoto Shimizubata, Shunsuke Minakuchi

**Affiliations:** 1 Department of Gerodontology and Oral Rehabilitation, Graduate School of Medical and Dental Sciences, Tokyo Medical and Dental University, 1-5-45 Yushima, Bunkyo, Tokyo 113-8549, Japan; tonprasong.gerd@tmd.ac.jp (W.T.); k.hatano.gerd@tmd.ac.jp (K.H.); makotobata@gmail.com (M.S.); s.minakuchi.gerd@tmd.ac.jp (S.M.); 2 Department of Microbiology, Nihon University School of Dentistry, 1-8-13 Kanda Surugadai, Chiyoda, Tokyo 101-8310, Japan; tamura.muneaki@nihon-u.ac.jp; 3 Department of Advanced Biomaterials, Graduate School of Medical and Dental Sciences, Tokyo Medical and Dental University, 1-5-45 Yushima, Bunkyo, Tokyo 113-8549, Japan; uo.abm@tmd.ac.jp (M.U.); wada.abm@tmd.ac.jp (T.W.); 4 Department of Cariology and Operative Dentistry, Graduate School of Medical and Dental Sciences, Tokyo Medical and Dental University, 1-5-45 Yushima, Bunkyo, Tokyo 113-8549, Japan; renatakahashi@hotmail.com

**Keywords:** surface pre-reacted glass ionomer (S-PRG) filler, nanofiller, tissue conditioner, ion release, acid buffering capacity, surface roughness, consistency, shore A0 hardness, detail reproduction, fungal adhesion

## Abstract

We aimed to evaluate the properties of a novel tissue conditioner containing a surface pre-reacted glass-ionomer (S-PRG) nanofiller. Tissue conditioners containing 0 (control), 2.5, 5, 10, 20, or 30 wt% S-PRG nanofiller or 10 or 20 wt% S-PRG microfiller were prepared. The S-PRG nanofillers and microfillers were observed using scanning electron microscopy. The ion release, acid buffering capacity, detail reproduction, consistency, Shore A0 hardness, surface roughness, and *Candida albicans* adhesion of the tissue conditioners were examined. The results indicated that the nanofiller particles were smaller and more homogeneous in size than the microfiller particles. In addition, Al, B, F, and Sr ions eluted from S-PRG were generally found to decrease after 1 day. Acid neutralization was confirmed in a concentration-dependent manner. The mechanical properties of tissue conditioners containing S-PRG nanofiller were clinically acceptable according to ISO standard 10139-1:2018, although the surface roughness increased with increasing filler content. Conditioners with 5–30 wt% nanofiller had a sublethal effect on *C. albicans* and reduced fungal adhesion in vitro. In summary, tissue conditioner containing at least 5 wt% S-PRG nanofiller can reduce *C. albicans* adhesion and has potential as an alternative soft lining material.

## 1. Introduction

Tissue conditioners have been clinically used as temporary soft lining materials for traumatized mucosa of denture wearers. Their cushioning effects, which include absorbing the bite force and reducing direct pressure on the denture-bearing area, are considered essential for promoting soft tissue healing. Plasticizers and solvents are important components of tissue conditioners, but they can leach out during material hardening. For these reasons, tissue conditioners are used to clinically modify dynamic impression materials. Conversely, due to the instability of these materials, storage may lead to deterioration, depending on the storage technique and time [1]. Moreover, the mechanical cleaning method has been reported to increase the roughness of the soft lining material [2], which promotes microbial accumulation.

Pathogenic microbes cause oral diseases by destroying hard tissue and/or generating soft tissue lesions. *Candida albicans* is an opportunistic fungus that plays a key role in oral candidiasis and denture stomatitis [3]. Several reports have indicated its high affinity toward the denture surface, which is potentially detrimental to the underlying mucosa [3,4]. This condition is especially serious in patients who are elderly, immunocompromised, suffering from underlying diseases, undergoing radiotherapy, or taking multiple medications [5]. To invade target cells, *C. albicans* exhibits fungal adhesin and undergoes a morphological transformation. Furthermore, a major virulence factor of *C. albicans* is the production of biofilms [6]. It was recently shown that denture-associated microbiomes are a contributing factor to respiratory infections, especially in immunocompromised patients [7].

Although tissue conditioner is routinely used as a temporary lining material, it could also become a plaque reservoir to cause the serious complications mentioned above. The incorporation of bioactive agents into tissue conditioners is considered an alternative way of suppressing oral pathogens. Tested bioactive agents include antimicrobials such as nystatin [8,9,10,11,12], chlorhexidine [12,13], fluconazole [10,14,15], ketoconazole [12], itraconazole [11], and clotrimazole [15]; natural extracts such as chitosan oligosaccharide [16], terpinen-4-ol [17], and cinnamaldehyde [17]; and inorganic agents such as ZnO/Ag nanoparticles [18], silver zeolite [19], magnesium oxide [20], and surface pre-reacted glass-ionomers (S-PRGs) [21]. However, several studies have reported that bioactive agents can degrade the performance of tissue conditioners.

As inorganic bioactive fillers, S-PRGs have the additional ability to release ions [22]. The released ions can exhibit a variety of bioactive behaviors, such as preventing fungal [23] and bacterial [24] adhesion, antibacterial activity [25], neutralizing acids [26], inhibiting demineralization [26], and enhancing remineralization [22]. Takakusaki and team [21] reported that S-PRG filler at 10 wt% or higher can reduce *C. albicans* adhesion. However, such a high filler volume negatively impacts the surface characteristic and mechanical properties of this novel tissue conditioner.

Compared to S-PRG microfillers comprised of microsized particles, recently developed nano-sized S-PRG fillers (nanofillers) are expected to be an improvement, because their smaller particle size and larger surface-to-volume ratio [27] suggest enhanced ion release and better mechanical properties. However, the properties of tissue conditioners containing S-PRG nanofillers have not been adequately examined.

This study aimed to investigate a novel tissue conditioner containing a S-PRG nanofiller, in terms of the chemical properties (ion release and acid buffering capacity), mechanical properties (consistency, detail reproduction, Shore A0 hardness, and surface roughness), and biological properties (*C. albicans* adhesion). The null hypothesis is that there is no difference in properties between tissue conditioners with and without the S-PRG nanofiller.

## 2. Materials and Methods

### 2.1. Morphological Observation of S-PRG Particles

The materials used in this study are listed in Table 1. For morphological observation, a thin layer of pure S-PRG nanoparticles or S-PRG microparticles was prepared on an aluminum stub with carbon tape. Excess particles were removed using an air-blowing technique. The specimens were coated with palladium and platinum nanoparticles using an automatic coating machine (Quick Auto Coater sc-701AT, Sanyu Electron, Tokyo, Japan). The morphologies of both types of particles were observed using scanning electron microscopy (SEM; Hitachi S-4500, Hitachi, Tokyo, Japan) at an accelerating voltage of 15 kV, an emission current of 10 µA, and a working distance of 15 mm.

### 2.2. Preparation of Tissue Conditioners

S-PRG nanoparticles or microparticles were incorporated into a tissue conditioner powder (Shofu, Kyoto, Japan) at various concentrations, as listed in Table 2. Tissue conditioners containing 2.5, 5, 10, 20, and 30 wt% S-PRG nanofiller (labeled as 2.5 Nano, 5 Nano, 10 Nano, 20 Nano, and 30 Nano, respectively) and 10 and 20 wt% S-PRG microfiller (labeled as 10 Micro and 20 Micro, respectively) were prepared by the manufacturer. All materials and specimens used in this study were stored at room temperature and away from direct sunlight. Following the manufacturer’s recommendation, the tissue conditioner powder with the S-PRG filler was gently mixed with the tissue conditioner liquid at room temperature while taking care to avoid forming air bubbles. The powder/liquid ratio was 1.2 g of powder per 1 mL of liquid. After complete mixing, disk-shaped tissue conditioner specimens were fabricated using a ring-shaped polycarbonate mold with double glass plates and clamped in place. Five minutes after mixing, the specimens were removed from the mold, and the absence of air bubbles was checked visually before further testing. A control sample with no S-PRG filler was also prepared in the same manner.

### 2.3. Ion Release

Tissue conditioner specimens with dimensions of 10 mm (diameter) × 1.6 mm (thickness) were prepared according to the protocol described in Section 2.2. After removal from the mold, each specimen was immediately immersed in 20 mL of ultrapure water in a conical polypropylene tube and maintained at 37 °C. The immersion solution was changed every 24 h for 7 days. When evaluating the ion release behavior at different particle sizes, the S-PRG microfiller and nanofiller were compared at pairs of the same percentages (10wt% or 20wt%) of S-PRG microfiller and nanofiller particles. The eluate was collected and stored in a refrigerator at 10 °C. The concentrations of released Al, B, and Sr ions were quantified by inductively coupled plasma atomic emission spectroscopy (ICP-AES, Spectro Arcos, Hitachi High-Technologies, Tokyo, Japan). A multi-element standard solution (100 ppm, XSTC-22, Seishin Trading, Hyogo, Japan) and Sr standard solution (1000 ppm, Nacalai Tesque, Kyoto, Japan) were used as reference. The concentrations of released F ions were measured using an ion meter (ORION 4STAR, Thermo Fisher, Tokyo, Japan) equipped with a fluoride ion electrode (9609 BNWP, Thermo Fisher), with reference to a fluoride ion standard solution (1000 ppm, Fujifilm Wako Pure Chemical Corp., Osaka, Japan). Four samples were tested for each of the experimental and control groups. The data were statistically compared using two-way repeated-measures analysis of variance (ANOVA) followed by a multiple comparison test (α = 0.05) with the R software package (R Foundation for Statistical Computing, Vienna, Austria).

### 2.4. Acid Buffering Capacity

Tissue conditioner specimens with dimensions of 12 mm (diameter) × 2.5 mm (thickness) were prepared according to the protocol described in Section 2.2. The specimens were immersed in 10 mL of a demineralizing solution (pH = 4.3) containing 2.8 mmol/L CaCl_2_, 2.8 mmol/L, NaH_2_PO_4_, and 50 mmol/L acetic acid. After 24 h of incubation at 37 °C, the specimens were removed from the solution. The pH change of the solution was measured with a pH meter (Waterproof pH 310 m, Oakton, Vernon Hills, IL, USA) that was calibrated using standard buffers at pH 4.01, 7.01, and 10.01. Five samples were tested for each experimental and control group. The data were statistically compared using the Shapiro–Wilk test followed by either one-way ANOVA with Tukey’s method or the Kruskal–Wallis test with Dunn’s test (α = 0.05) using the R software package.

### 2.5. Detail Reproduction

Detail reproduction experiments were performed according to ISO standard 10139-1:2018 [28]. First, a metal test block with three vertical lines on the surface (20, 50, and 75 µm in width) and a ring mold were immersed in distilled water at 37 °C. After 15 min, they were taken out of the distilled water, and excess moisture was removed using an airstream. The metal ring was seated vertically on the test block, with the three vertical lines within the ring, to generate a specimen-forming cavity. A tissue conditioner powder/S-PRG filler/liquid mixture was freshly prepared (see Section 2.2) and gradually poured into the cavity. A glass plate on a polyethylene sheet was immediately pressed against the top of the ring mold. At 60 s after complete mixing, the whole assembly was immersed in distilled water maintained at 37 °C for 48 h. After the incubation period, the detail reproduction of the three vertical lines was evaluated using a stereo microscope (TW180, Wraymer, Raymer, Osaka, Japan). One sample was tested for each of the experimental and control groups. The data were interpreted using the criteria of ISO standard 10139-1:2018 [28].

### 2.6. Consistency

Consistency experiments were performed following ISO standard 10139-1:2018 [28]. The top surface of a glass plate (100 mm × 100 mm) was covered with a polyethylene sheet. A tissue conditioner powder/S-PRG filler/liquid mixture was freshly prepared (see Section 2.2). Thirty seconds after complete mixing, 2 mL of the mixture was dispensed using a plastic syringe (Terumo, Tokyo, Japan) in the center of the polyethylene sheet. The assembly was transferred to an incubator maintained at 37 °C. The surface of the material was then covered with a polyethylene sheet and glass plate (100 g in weight). At 120 s after complete mixing, a 1 kg weight was placed atop the upper glass plate for 300 s. Subsequently, the weight and upper glass plate were removed. The maximum and minimum diameters of the tissue conditioner were measured using a ruler with subdivisions of 1.0 mm, and the average diameter was calculated. Four samples were tested for each of the experimental and control groups. The data were statistically compared using the Shapiro–Wilk test followed by either one-way ANOVA with Tukey’s method or the Kruskal–Wallis test with Dunn’s test (α = 0.05) using the R software package.

### 2.7. Shore A0 Hardness

Shore A0 hardness experiments were performed according to ISO standard 10139-1:2018 [28]. Tissue conditioner specimens were prepared using a metal mold (diameter: 50 mm, thickness: 8 mm) with two glass plates. Fifteen minutes after mixing, the specimens were immersed in 100 mL of water and kept in an incubator maintained at 37 °C for 2 h. The specimens were then removed from the metal mold and placed on a glass plate. A durometer (UF.Shore, Ueshima Seisakusho, Tokyo, Japan) was manually deployed. After verifying that the device surface was parallel to the specimen surface, the indenter foot of the device was slowly pressed onto the specimen surface. The values were recorded after 5 s of loading, and 5 points were measured within 3 min. Then, the specimens were returned to water maintained at 37 °C and kept for 7 days. After the second incubation period, the other side of the specimen was measured using the same protocol. Three samples were tested for each of the experimental and control groups. The data were statistically compared using two-way repeated measures ANOVA followed by multiple comparison tests (α = 0.05) using the R software package.

### 2.8. Surface Roughness

Tissue conditioner specimens with dimensions of 12 mm (diameter) × 2.5 mm (thickness) were prepared according to the protocol described in Section 2.2. The specimens were immersed in 20 mL of artificial saliva containing 0.2 mmol/L CaCl_2_, 0.2 mmol/L KH_2_PO_4_, 1 mmol/L NaCl, 1 mmol/L C_2_H_3_NaO_2_, and 2% NaN_3_. After incubation at 37 °C for 24 h, the specimens were immediately removed and dried using an air-blowing technique. Ten areas were randomly selected on each specimen and observed using confocal laser scanning microscopy (LEXT OLS4100, Olympus, Tokyo, Japan) at 50 µm cutoff and 50× magnification. Five samples were examined for each of the experimental and control groups. The data were statistically compared using the Shapiro–Wilk test followed by either one-way ANOVA with Tukey’s method or the Kruskal–Wallis test with Dunn’s test (α = 0.05) using the R software package.

### 2.9. Candida albicans Adhesion

#### 2.9.1. Specimen Preparation

Tissue conditioner specimens with dimensions of 10.0 mm (diameter) × 1.6 mm (thickness) were prepared according to the protocol described in Section 2.2. Three samples were prepared for each of the experimental and control groups. Clean polypropylene disks (diameter: 12 mm, thickness: 0.5 mm) that had been disinfected using 70% ethyl alcohol for a few seconds were used as a base of the specimen to prevent unexpected attachment during the experimental procedure.

#### 2.9.2. Saliva Coating

The specimens were placed in a 24-well plate and pre-rinsed twice with 1 mL of 0.87% NaCl. After removing the saline solution, the specimens were immersed in 1 mL of artificial saliva (0.381 g NaCl, 0.213 g CaCl_2_·2H_2_O, 1.114 g KCl, 0.738 g KH_2_PO_4_, and 1.1 g mucin in 1 L distilled water, pH 7) under agitated conditions (BR-12FH, BioShaker, Saitama, Japan) at 37 °C for 30 min.

#### 2.9.3. *Candida albicans* Preparation and Adhesion

*C. albicans* (ATCC18804) was cultured for 12 h in Sabouraud glucose (SG) media at a constant temperature of 37 °C. The yeast suspension was centrifuged and washed twice with 0.87% NaCl solution. The yeast suspension was quantified to 10^7^ cells/mL using a spectrophotometer (U1100, Hitachi, Tokyo, Japan) at an optical density (OD) of 550 nm. The prepared tissue conditioner specimens with saliva coating were treated with 1 mL of yeast suspension under agitated conditions at 37 °C for 1 h.

#### 2.9.4. Fluorescent Staining and Observation

After inoculation, the specimens were removed from the yeast suspension, washed twice with 0.87% NaCl to remove unattached cells, and transferred to a new plate. The adhering cells were stained using the Live/Dead FungaLight^TM^ yeast viability kit (Lot No. L34529, Molecular Probes, Eugene, OR, USA) by adding 500 µL of 0.87% NaCl, 1 µL of SYTO-9, and 1 µL of propidium iodide (PI) into the 24-well plate, followed by incubation for 30 min in the dark at room temperature. Then, the specimens were mounted in a 35 mm diameter non-coated culture dish (GlassBottomDish, Matsunami Glass IND., LTD, Osaka, Japan). Cell adhesion was observed using confocal scanning laser microscopy (FV10i, Olympus Corporation, Tokyo, Japan) under the z-stack mode at 10× magnification. Three areas were randomly chosen on each specimen for observation. Yeast suspension was used as the positive control. After images were obtained for an area of 1.27 mm × 1.27 mm, the number of adhering cells was quantified using ImageJ software (U.S. National Institutes of Health, Bethesda, MD, USA). The red-stained cells were considered dead cells, and they were subtracted from the total cell number to calculate the number of living cells. The dead/living cell ratio and number of living adherent cells were analyzed. The data were statistically compared using the Shapiro–Wilk test followed by either one-way ANOVA with Tukey’s method or the Kruskal–Wallis test with Dunn’s test (α = 0.05) using the R software package.

## 3. Results

### 3.1. Morphology of S-PRG Fillers

**Figure 1:**
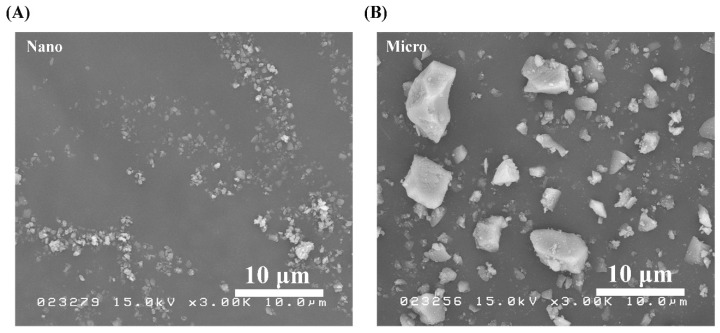
SEM images of pure S-PRG (**A**) nanoparticles and (**B**) microparticles. Polygonal particles were observed in both cases.

SEM micrographs of the S-PRG nanoparticles and microparticles were obtained under the same observation conditions and shown in Figure 1A,B, respectively. Between the two fillers, the nanoparticles were confirmed to have a smaller size. In addition, both types of particles had irregular microstructures, while nonhomogeneous particle sizes were observed in the microfiller.

### 3.2. Ion Release

**Figure 2:**
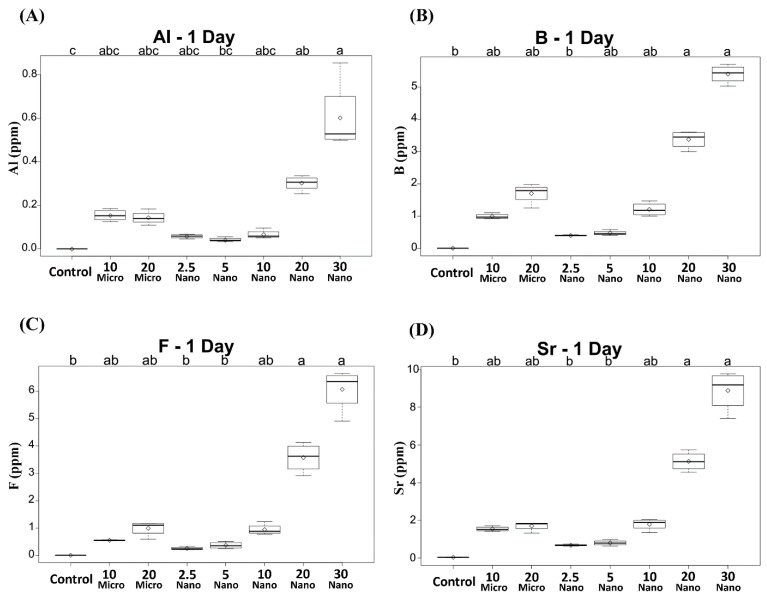
Concentrations of (**A**) Al, (**B**) B, (**C**) F, and (**D**) Sr ions released after 1 day from tissue conditioners incorporating different ratios of S-PRG filler and the control. Different letters above the boxplots indicate statistically significant differences.

**Figure 3:**
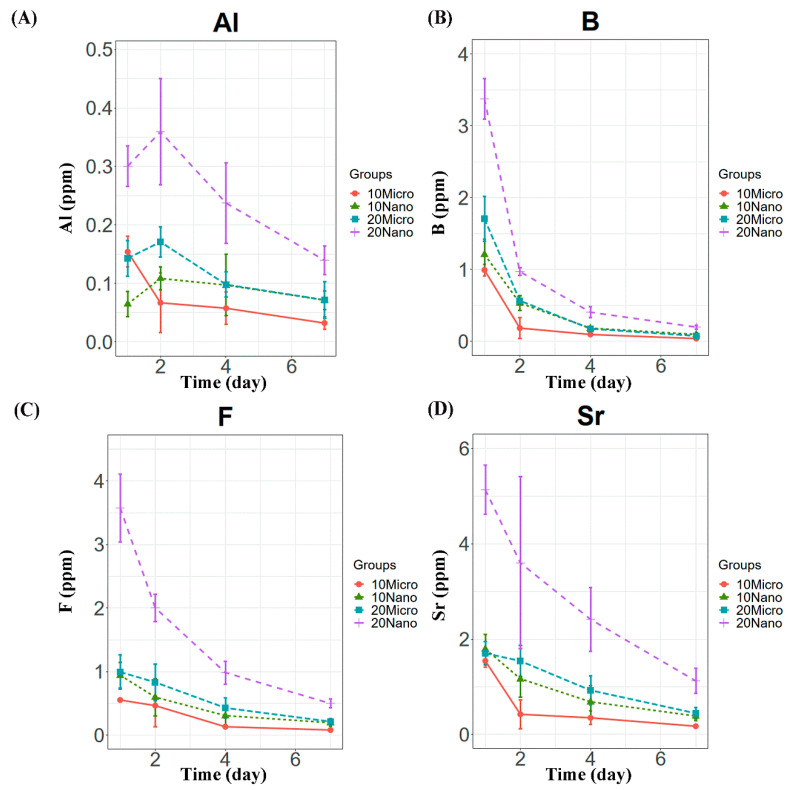
Ion release behavior from tissue conditioner specimens containing 10 and 20 wt% microfiller and nanofiller particles on days 1, 2, 4, and 7. Panels (**A**–**D**) show the Al, B, F, and Sr concentrations, respectively.

All the specimens containing the S-PRG filler released detectable concentrations of Al, B, F, and Sr ions. Compared to the control, only the conditioners with 20 or 30 wt% nanofiller exhibited significantly higher ion release on day 1 (*p* < 0.01), as shown in Figure 2. The levels of released B, F, and Sr were the highest on day 1 and decreased in subsequent days (Figure 3). However, Al release showed no significant difference between days 1 and 2 (*p* > 0.05), as shown in Figure 3A. Among the specimens containing the microfiller and nanofiller at the same weight ratio, 20 Nano released significantly higher concentrations of Al, B, F, and Sr than 20 Micro at all measurement times (*p* < 0.05). In contrast, there was no statistically significant difference in the concentration of Al ions released from 10 Nano and 10 Micro after day 1 (*p* > 0.05). The concentration of F ions released from 10 Nano and 10 Micro presented no statistically significant difference on any day (*p* > 0.05), except day 7 (*p* = 0.0098). In addition, there was no statistically significant difference in the concentration of B and Sr released from 10 Nano and 10 Micro at all measurement times (*p* > 0.05).

### 3.3. Acid Buffering Capacity

**Figure 4:**
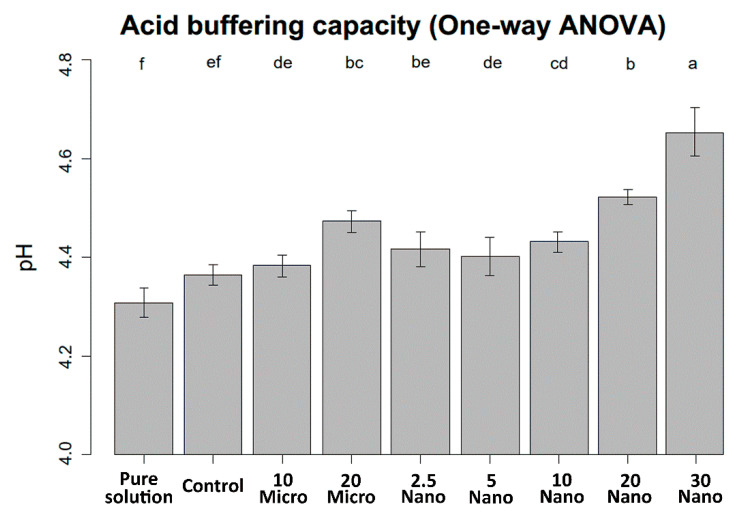
pH levels of the demineralizing solution after immersing the experimental and control specimens for 24 h. Different letters above the bars indicate significant differences.

The mean values and standard deviations of the pH changes are shown in Figure 4. The pH of the demineralizing solution increased with increasing S-PRG filler content. One-way ANOVA indicates that the pH value was not significantly different between the control specimen (tissue conditioner without S-PRG) and the pure demineralizing solution (4.31 ± 0.03 and 4.36 ± 0.02, respectively, *p* = 0.11). Hence, the tissue conditioner has no acid buffering capacity before S-PRG addition. The 30 Nano specimen induced the highest increase in pH after immersion for 24 h (4.65 ± 0.05, *p* < 0.001). However, there were no significant differences in acid neutralization capacity between samples with the microfiller and nanofiller at the same concentration (*p* > 0.05).

### 3.4. Detail Reproduction

The results of the detail reproduction experiments are presented in Table 3. After 2-day incubation, the 20, 50, and 75 µm continuous lines were observed on all specimens including the controls. Since the ISO specification only requires the detection of the 75 µm line [28], all specimens met the ISO standard.

### 3.5. Consistency

Table 3 shows the mean values and standard deviations of the consistency data. All specimens including the control had diameters in the range of 25 mm to less than 60 mm and are therefore classified as class 1 (medium flow) according to ISO specification 10139-1:2018 [28]. However, the consistency improved as the amount of S-PRG filler increased. The Kruskal–Wallis test followed by Dunn’s test (α = 0.05) shows that two compositions (20 Micro at 53.40 ± 0.50 mm and 30 Nano at 54.68 ± 2.18 mm) have significantly higher consistency (*p* < 0.01) than the control (47.30 ± 1.08 mm).

### 3.6. Shore A0 Hardness

Table 3 shows the mean values and standard deviations of the Shore A0 hardness at 2 h and 7 days. When measured at 2 h, all experimental and control groups had hardness values of less than 30 and are therefore classified as type b (extra soft) following ISO specification 10139-1:2018 [28]. At 7 days, all experimental groups had mean hardness values of less than 60, which complies with the ISO standard. From statistical analysis using a two-factor repeated-measures ANOVA, the mean hardness at day 7 was significantly higher than that at 2 h for all experimental and control groups (*p* < 0.01). The 30 Nano has the lowest hardness value at both 2 h and day 7 (*p* < 0.001). When comparing the samples with the same concentration of S-PRG filler but different particle sizes, the hardness of 20 Nano was significantly lower than that of 20 Micro at 2 h and 7 days (*p* < 0.01). Meanwhile, there were no significant differences between 10 Nano and 10 Micro at either measurement time (*p* > 0.05).

### 3.7. Surface Roughness

**Figure 5:**
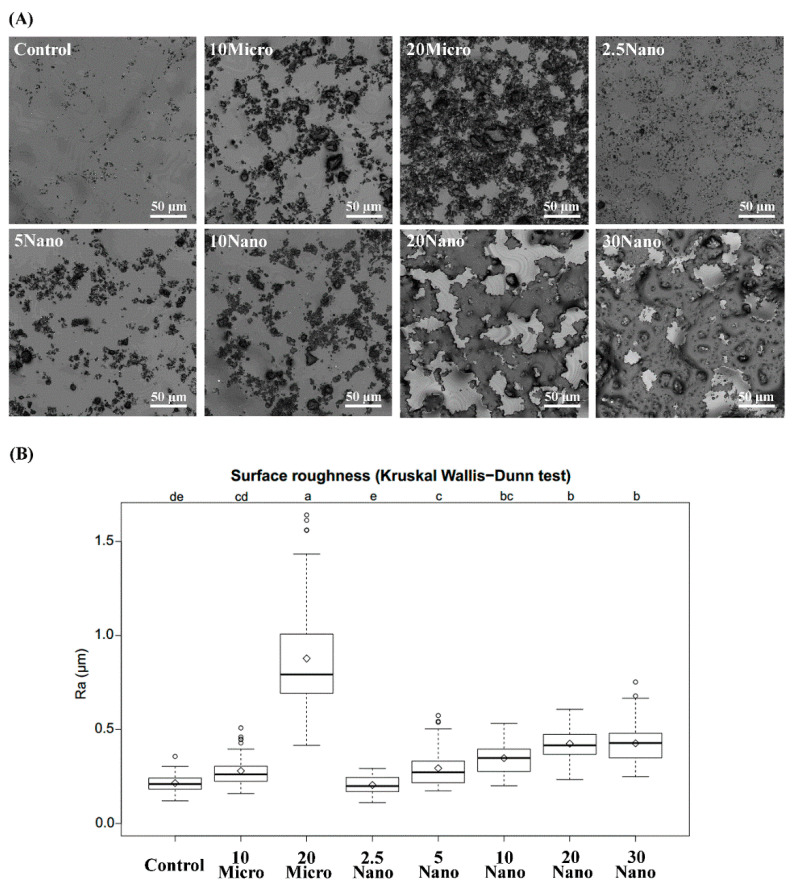
(**A**) Laser scanning micrographs illustrating the surface detail of different tissue conditioners. (**B**) Boxplots of surface roughness measured for different tissue conditioner specimens. Different letters above the plots indicate significant differences.

Figure 5A contains representative images of the S-PRG filler distribution on the tissue conditioner surface taken by 3D laser microscopy. Figure 5B shows boxplots summarizing the mean values and standard deviations of the surface roughness. The surface roughness tended to increase with an increasing amount of the S-PRG filler. Kruskal–Wallis tests followed by Dunn’s test indicated that 20 Micro had the highest surface roughness (0.88 ± 0.33 µm), while 20 Nano had a significantly (*p* < 0.01) smoother surface (0.43 ± 0.08 µm). However, there was no significant difference (*p* > 0.05) between the surface roughness of 10 Micro (0.28 ± 0.08 µm) and 10 Nano (0.35 ± 0.09 µm). Moreover, no statistically significant difference (*p* > 0.05) was found between 20 Nano (0.43 ± 0.08 µm) and 30 Nano (0.43 ± 0.11 µm).

### 3.8. Candida albicans Adhesion

**Figure 6:**
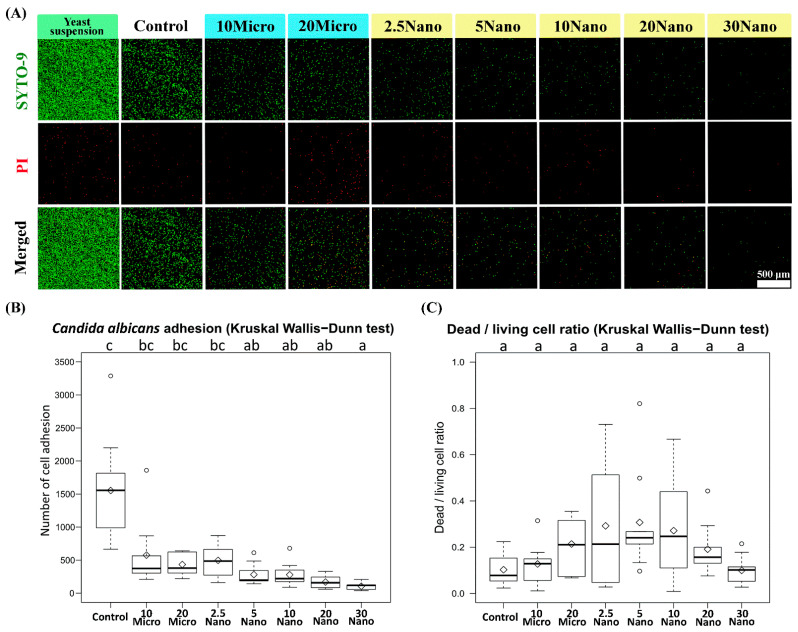
(**A**) Confocal laser scanning fluorescence micrographs showing *Candida albicans* cell adhesion on tissue conditioner specimens. The red-colored cells were stained using propidium iodide (PI) and considered dead. (**B**) Number of cell adhesion and (**C**) dead/living cell ratio on the specimens. The number of living cells (colored green) was calculated as the total cell number minus the dead cell number.

Figure 6A presents confocal laser scanning fluorescence micrographs of the samples after inoculation in a *C. albicans* yeast suspension. We found that *C. albicans* (ATCC18804) diffusely adhered onto the surface of the control tissue conditioner (without S-PRG filler) and experimental groups. The Kruskal–Wallis test followed by Dunn’s test indicated a statistically significant suppression of fungal adhesion when the specimen contained at least 5 wt% S-PRG nanofiller (281.33 ± 166.20 cells) as compared to the control (1553.89 ± 829.97 cells, *p* < 0.01). Meanwhile, the suppression was not observed for 2.5 Nano (496.33 ± 236.23 cells), 10 Micro (575.22 ± 521.09 cells), or 20 Micro (433.89 ± 164.90 cells, *p* > 0.05), as shown in Figure 6B. The strongest suppression occurred in 30 Nano (104.78 ± 54.60 cells), which reduced fungal adhesion by approximately 15-fold compared to the control. There was no statistically significant difference (*p* > 0.05) in the dead/living cell ratio between the experimental groups and the control group, as shown in Figure 6C. Thus, the nanofiller was confirmed to have a sublethal capacity to reduce the adhesion of *C. albicans* on the tissue conditioner surface in a concentration-dependent manner.

## 4. Discussion

The present study revealed that the addition of S-PRG nanofiller improves the chemical and biological properties of the studied tissue conditioner. Therefore, the null hypothesis is rejected. Meanwhile, the mechanical properties change slightly but conform to the full requirement of ISO standard.

S-PRG filler is considered a multifunctional bioactive glass because it can release several types of ions including Al, B, F, Na, Si, and Sr. Several studies have reported the effect of these ions on the tooth structure. Murayama et al. [29] reported that F ions released from a coating material containing a S-PRG filler inhibited demineralization. Ogawa et al. [22] found that Al, B, and Sr ions can be incorporated into human tooth enamel after immersion in an eluate of S-PRG filler. A synergistic effect between F and Sr ions on enamel remineralization was confirmed [30]. In addition to the enamel structure, dentin was also found to benefit from S-PRG filler. Shiiya et al. [31] reported that S-PRG filler delayed the demineralization of bovine dentin after acidic challenge. Fujimoto et al. [32] mentioned that an acidic environment had a limited negative influence on ion release. However, enhanced ion release was observed when immersing S-PRG filler in a larger amount of solution. In addition, several studies reported that after immersing pure S-PRG filler in solution, the concentration of B ions was about 10-fold higher than that of Sr ions [22,31]. In contrast, other researchers observed a lower B/Sr ratio after S-PRG filler was added to different materials, including tissue conditioners [21], denture base resins [33], composite resins [24,34,35], coating materials [36], and dental sealants [37,38,39,40]. Even though the cause of suppressed ion release from these materials is unclear, it is possible that the chemical interactions and/or different solubilities of each material affect the manner of ion release. Similar to these previous reports, we observed suppressed ion release after the S-PRG nanofiller was mixed in the tissue conditioner.

Considering the clinical applications, we simulated routine tissue conditioner usage by changing the immersion solution every 24 h for up to 7 days, which is the recommended maximum period for the clinical use of tissue conditioners according to the ISO standard. B, F, and Sr ions were rapidly released on day 1, while the released amounts decreased significantly over time. Therefore, 1-day treatment may be the optimal period for maximizing the benefit of the S-PRG filler in the tissue conditioner.

Several studies have shown the relationship between ion release and acid-buffering capacity. Fujimoto et al. [32] found that after 24 h of mixing a S-PRG filler with lactic acid solution, the pH became more neutral. Nakajo et al. [41] reported that F, Si, and Al ions from the elution of glass ionomer cement enhanced acid neutralization and inhibited acid production from oral streptococci. In our study, both the ion release and acid neutralization were observed in a concentration-dependent manner, indicating a possible correlation between the concentration of the S-PRG filler and acid neutralization.

The basic mechanical properties of the tissue conditioner were investigated according to ISO specification 10139-1:2018. In the consistency tests, we found increased fluidity when the specimens incorporated more S-PRG filler, which agrees with an observation from another study [21].

Impression-taking is a useful function of tissue conditioners. Nili and Abdoulhamidi [42] indicated that prolonged storage in water can strongly affect the surface quality and detail reproduction of different brands of tissue conditioners on a gypsum model. However, we found no difference in detail reproduction between tissue conditioners with and without the S-PRG nanofiller or microfiller at various concentrations, even after 7 days of storage. Therefore, detail reproduction is not affected by the addition of ≤20 wt% S-PRG microfiller or ≤30 wt% S-PRG nanofiller. Due to its smaller particle size, as shown in Figure 1, the nanofiller may have a better ability than the microfiller to blend and flow with the polyethyl methacrylate matrix.

The Shore A0 hardness test examines the viscoelasticity of the test material. Jones et al. [43] mentioned that leaching of the plasticizer and evaporation of the alcohol caused physical changes in the tissue conditioner. In addition, Urban et al. [44] reported that the hardness of tissue conditioners can increase during storage, which could cause discomfort to the patient, such as when chewing. The results here are similar to those in previous studies [15,17,44]. However, we found that adding 20 wt% of the microfiller into the tissue conditioner resulted in significantly higher hardness than that when the nanofiller was added at the same weight ratio. The possible reason is that the quantity and particle size of the filler affected the surface hardness. Nevertheless, our tests based on the ISO standard confirmed that the tissue conditioner containing S-PRG had clinically acceptable mechanical properties.

The surface roughness of a material is strongly correlated with microbial adhesion [45,46]. Takakusaki et al. [21] concluded that increasing the amount of S-PRG filler in a tissue conditioner increased its surface roughness. Our measurements here showed a consistent trend. Our morphological observation revealed that the S-PRG microfiller particles had a nonhomogeneous size, whereas the S-PRG nanofiller particles were smaller and more homogeneous in size. Therefore, the tissue conditioner containing the nanofiller had a significantly smoother surface than that containing the microfiller, with both fillers at 20wt%. From these results, we hypothesized that not only the quantity but also the quality of the S-PRG filler affects the surface roughness of the tissue conditioner. Besides the physical characteristics of the material, the storage technique [1] and time [47] are also important factors that affect surface roughness. Hong et al. [1] recommend storing dynamic impressions in the air to prevent changes in surface roughness. Considering the physiological stability of the material and to prevent ions from being released into the storage medium, we suggest storing the S-PRG filler containing tissue conditioner in the air. However, further investigation is required to determine the optimal storage technique.

The biological effects of S-PRG fillers have been studied for several types of oral pathogens. Nomura et al. [25] reported that the eluate of the S-PRG filler has a protective effect against *Streptococcus mutans*, an important microbe for inducing carious lesions, through downregulation of the *pdh* and *glg* operons that are related to bacterial sugar metabolism. Moreover, the eluate of S-PRG filler reduced the density and thickness of the formed biofilm. Miki et al. [35] found that ions released from a resin composite play an important role in inhibiting the growth of *S. mutans.* Another organism of interest is *C. albicans*, which is considered an opportunistic pathogen associated with oral diseases. Pointer et al. [48] reported that 0.1% boric acid suppressed the hyphal transformation of *C. albicans*. Takakusaki et al. [21] showed that a S-PRG filler containing tissue conditioner reduced the colony-forming units (CFUs) of *C. albicans* in a dose-dependent manner.

This study focused on the adhesion phase of *C. albicans*, which is the initial pathological phase of candidiasis before colonization and biofilm formation. McCall et al. [49] demonstrated that *C. albicans* begins to attach during the first 2 h of the biofilm formation process. Djimeli et al. [50] presented the following qualitative stages of cell adhesion based on the fungal growth pattern: a lag of growth phase (0–5 h), exponential growth phase (5–13 h), stationary growth phase (13–22 h), and decline of growth phase (from 22 h onward). In this study, we experimentally simulated *C. albicans* adhesion on the tissue conditioner surface over 12 h of incubation, in order to observe the effect of the S-PRG filler during the active phase of the fungus. Moreover, confocal laser microscopy was employed for direct observation. The S-PRG nanofiller contained in the tissue conditioner had a sublethal effect on *C. albicans* and reduced the number of living cell adhesions. However, the antifungal adhesion mechanism of S-PRG filler is not completely understood. McCall et al. [50] indicated that the deletion of several transcription factors of adhesin proteins (Δ*efg1*, Δ*bcr1*, Δ*als1*, *and Δals3*) reduced the initial rates of fungal attachment. Tamura et al. [23] reported that the elution of the S-PRG filler induced oxidative stress in *C. albicans* by reducing hydrogen peroxide and catalase, which have been shown to act as antioxidants. The elution also suppressed secreted aspartyl proteinase, which plays an important role in the cell adhesion and hyphal transformation of *C. albicans*. It is possible that the ion release and acid neutralization behavior of the S-PRG filler can interrupt some of the above processes.

Although adding the S-PRG nanofiller into the tissue conditioner altered the surface characteristics of the material, there was clearly a less negative effect compared to the S-PRG microfiller. In addition, the inhibition of initial pathogen activity compensated for the roughened surface morphology by retarding pathogen adhesion/biofilm growth. From our findings, the addition of ≥5 wt% S-PRG nanofiller reduces the number of fungal cells on the surface of the tissue conditioner and may delay colonization and biofilm formation. These effects should be examined in additional animal and clinical studies to identify the effects of abrasion from brushing, storage technique, cytotoxicity, etc. The present in vitro study did not consider many factors such as the components of real saliva, oral pH, patient cleaning behavior, and duration of denture usage. Nevertheless, the results indicated that the S-PRG nanofiller-containing tissue conditioner is a potential alternative soft lining material for immunocompromised patients in the future.

## 5. Conclusions

Despite the limitations of an in vitro study, our results clearly demonstrated the concentration-dependent ion releasing ability and acid buffering capacity of a tissue conditioner containing S-PRG nanofillers, with acceptable mechanical properties as measured by ISO standards. Although increasing the filler content somewhat compromised the surface characteristics of the material, there was a strong preventive effect against candidiasis when at least 5 wt% S-PRG nanofiller was incorporated.

## Figures and Tables

**Table 1:** Trade name, manufacturer, composition, and batch code of materials used in this study.

Material	Trade Name	Composition	Manufacturer	Batch Code
Tissue conditioner (powder)	Shofu tissue conditioner II	Polyethyl methacrylate	Shofu, Kyoto, Japan	1208201
Tissue conditioner (liquid)	Shofu tissue conditioner II	Di-n-butyl sebacate and absolute ethanol	Shofu	111847
S-PRG microfiller	-	Pure S-PRG microfiller	Shofu	091901
S-PRG nanofiller	-	Pure S-PRG nanofiller	Shofu	091902

**Table 2:** Composition of tissue conditioner samples in the experimental and control groups (with and without S-PRG filler, respectively).

Groups	S-PRG Filler		Tissue Conditioner Powder, wt%
	Particle Size	wt%	
10 Micro	Micro	10	90
20 Micro	Micro	20	80
2.5 Nano	Nano	2.5	97.5
5 Nano	Nano	5	95
10 Nano	Nano	10	90
20 Nano	Nano	20	80
30 Nano	Nano	30	70
Control	-	0	100

**Table 3:** Consistency, detail reproduction, and Shore A0 hardness of various tissue conditioners.

Groups	Consistency (mm)		Detail Reproduction (µm)	Shore A0 Hardness			
				2 h		7 Days	
	Mean *	SD		Mean	SD	Mean	SD
**Control**	47.30 ^c^	1.08	20, 50, 75	3.47	0.74	7.87	0.91
**10 Micro**	52.73 ^abc^	0.44	20, 50, 75	3.67	0.62	6.07	0.46
**20 Micro**	53.40 ^ab^	0.50	20, 50, 75	3.60	0.50	5.47	0.64
**2.5 Nano**	48.90 ^abc^	0.59	20, 50, 75	4.33	0.49	7.80	0.77
**5 Nano**	48.55 ^bc^	0.29	20, 50, 75	4.20	0.56	7.53	0.74
**10 Nano**	49.40 ^abc^	0.46	20, 50, 75	4.13	0.64	6.13	0.74
**20 Nano**	50.85 ^abc^	0.82	20, 50, 75	2.67	0.62	4.07	0.70
**30 Nano**	54.68 ^a^	2.17	20, 50, 75	1.0	0.00	2.00	0.53

The requirements of ISO 10139-1:2018 standard: *Consistency test*; 25 mm ≤ diameter < 60 mm (class 1, medium flow), 60 mm ≤ diameter < 100 mm (class 2, high flow). *Detail production test*; the reproduction should be presented at least 75 µm. *Shore A0 hardness test*; at 2 h, 30 < Shore A0 ≤ 50 (type A, soft), Shore A0 ≤ 30 (type B, extra soft). At day 7, the hardness value should not be higher than 60 [28]. * Different superscript letters indicate statistically significant differences.

## Data Availability

The data presented in this study are available on request from the corresponding author.
